# Omicron: increased transmissibility and decreased pathogenicity

**DOI:** 10.1038/s41392-022-01009-8

**Published:** 2022-05-07

**Authors:** Gábor Bálint, Barbara Vörös-Horváth, Aleksandar Széchenyi

**Affiliations:** Institute of Pharmaceutical Technology and Biopharmacy, University of Pécs, Faculty of Pharmacy, 7624 Pécs, Rókus u. 2, Pécs, Hungary

**Keywords:** Infectious diseases, Health care

**Fig. 1:**
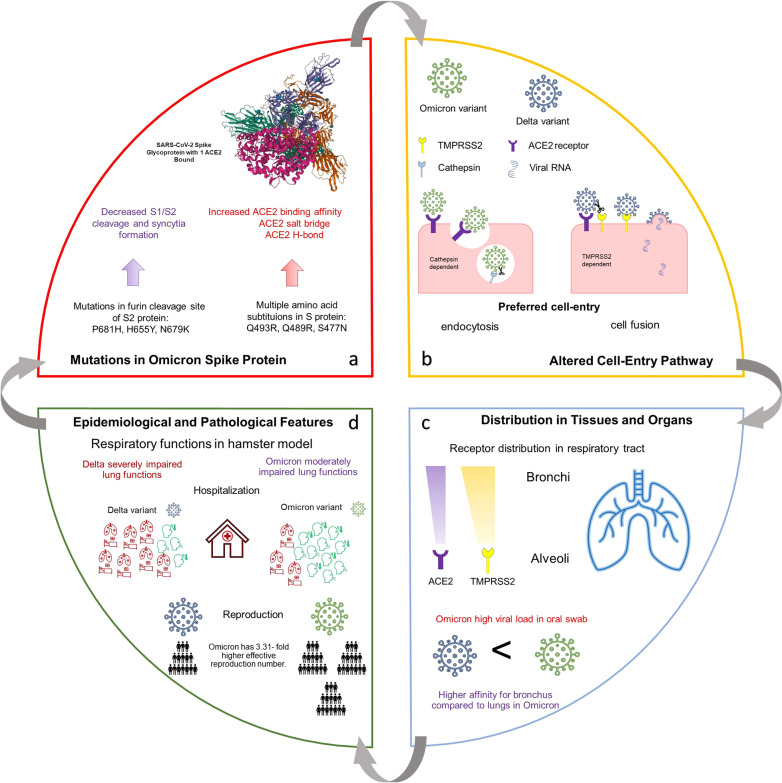
Schematic presentation of main epidemiological and virological factors interdependences: **a** Omicron viral spike protein mutations and their consequences. **b** Altered cell entry pathway caused by higher binding affinity toward ACE2 receptors and lack of S1/S2 cleavage site. **c** Receptor distribution and expression in the respiratory tract cells. **d** Comparison of epidemiological and pathological properties of Delta and Omicron variants from Epidemiological data and in vivo hamster model

Three research papers were published recently to compare the virological properties of the Omicron variant with the earlier variants of concern (VOC).^1–3^ The SARS-CoV-2 B.1.1.529 variant, Omicron, was first detected in South Africa in October 2021 and has spread rapidly; within three months, it appeared in more than 87 countries.^4^ During the surveillance of the epidemiological data and mutations of earlier COVID-19 variants and Omicron, significant differences were revealed mainly in the reproduction rate and hospitalization. Although a modest severity can be observed in the case of Omicron, its 3,31-fold higher transmissibility^2^ (Fig. 1d) than Delta variant and increased resistance to antiviral immunity^1^ represents a global epidemic threat.

Comparing the earlier SARS-CoV-2 variants with Omicron, it bears more mutations in its Spike protein, of which six in the S2 region are unique. Three mutations in the furin cleavage site region (P681H, H655Y, N679K) decrease S1/S2 cleavage, fusogenicity, and syncytia formation associated with pathogenesis. The multiple amino acid substitutions presumably cause the increased ACE2 binding affinity of Omicron in its S-protein, including Q493R, Q489R, and S477N (Fig. 1a). These mutations cause enhancement of ACE2 binding via the formation of ACE2 salt bridge and ACE2 H-bond.^2^ Analysis using advanced structure determining methods such as cryo-electron microscopy and X-ray crystallography confirmed these results and directly connected novel mutations and new chemical interaction sites.^5^

The ACE2 affinity was determined experimentally, using biolayer interferometry. Omicron RBD displayed a threefold higher binding affinity for ACE2 compared to Wuhan-HU-1 and Delta.^1^ This finding was confirmed by ACE2 antibody titration on cells transfected with a full-length spike.^1^ The receptor affinity and the receptor expression in cells affect viral tropism, which was examined in detail.

Immunohistochemical staining of ex vivo human tissue samples indicated higher ACE2 expression in bronchus than in lung, while analysis of mRNA expression levels indicated a similar distribution of TMPRSS2.^3^ Single-nuclei RNA sequencing performed on human lung tissues showed lower expression of TMPRSS2 in the trachea compared to alveoli.^1^ ACE2 expression was lower than TMPRSS2, although slightly elevated in specific cell types, such as AT1, AT2, and club cells.^1^ qPCR showed higher TMPRSS2 mRNA expression in lung parenchyma compared to upper airway bronchial tissue samples.^1^ In replication studies using multiple cell cultures, higher TMPRSS2 expression was revealed to favor the growth of delta variant, which relates to the impaired cleavage of the Omicron spike protein.^3^

In the VeroE6 cell model, TMPRSS2 expression proved to be favorable in the case of all three variants, but the magnitude of the difference compared to the non-TMPRSS2 expressing culture immensely varied, 100–1000-fold in Delta, 3–100-fold for Omicron and tenfold for WT.^3^ According to the AUC curves, Omicron replicated slower than WT and Delta.^3^ Further investigation on VeroE6/T2 cell culture using serin protease inhibitor camostat mesylate blocking the TMPRSS2 dependent cell entry pathway and E64d cathepsin inhibitor revealed significant dependence of Delta on TMPRSS2 and an even more pronounced dependence of Omicron on cathepsin mediated endocytosis.^3^ Pseudotyped virus assay using 3D lower airway organoids gallbladder organoids further supported the claim that the Omicron variant almost exclusively utilizes the cathepsin-dependent endocytic pathway while the Delta variant enters the cell by endocytosis and TMPRSS2 dependent fusion^1^ (Fig. 1b). Further investigation of the phenomenon by the blockade of the TMPRSS2 dependent and the cathepsin-dependent pathway also supported the theory.^1^ Studies on A549 cells showed no replication in ACE2 non-expressing culture.^2^

Rigel et al. compared the cellular growth of Omicron (strain TY38-873), D614G bearing (B.1.1 lineage, strain TKYE610670), and Delta isolates (B.1.617.2 lineage, strain TKYTK1734). No significant difference was observed in VeroE6/TMPRSS2 and primary human epithelial cells, while in Vero, Calu-3, A549-ACE2, HeLa-ACE2/TMPRSS2, the growth of the Delta exceeded the Omicron variant.^3^ Meng et al. examined the replication kinetics of Omicron and Delta in primary human nasal epithelial 3D cultures finding similar rates, while for Calu-3 lung cells, Caco-2 and HeLa overexpressing ACE2 and TMPRSS2^1^ Delta showed significantly higher replication rate. Contradictory to the previous two studies, it was found that Omicron replicated significantly faster in comparison to WT and the Delta variant at 24 h.p.i., 48 h.p.i. reaching over 70-fold difference, using TCID50 assay in ex vivo human bronchus tissue cultures.^3^ Although at the72 h.p.i. there was no significant difference between Delta and Omicron; the replication of the WT slowed down. Under similar conditions, lung samples infected by the Omicron variant showed reduced replication compared to WT and Delta.^3^

In VeroE6/TMPRSS2 cell culture, the growth kinetics showed similarities, but the Delta variant formed larger syncytia compared to B.1.1, while the Omicron infection can be described by weak syncytia formation^2^ due to attenuated fusogenicity. Cell-based fusogenicity assay recorded the lowest spike protein expression in case of Delta infection and the highest fusogenicity. Omicron infected cells expressed S-protein with a comparable amount to the parental variant, albite showing highly reduced fusogenicity,^2^ which may be directly related to decreased pathogenicity. In vivo studies using the hamster model confirmed that Omicron possesses decreased pathogenicity, investigating lung function parameters: enhanced pause, total expiratory time, and subcutaneous oxygen saturation.^3^ Detailed examination of respiratory organs in infected hamsters revealed extensive inflammatory nodule formation for Delta and B.1.1. and sporadic for Omicron (Fig. 1d).

Immunohistochemical analysis of viral N-protein revealed low infectivity of all SARS-COV-2 isolates tested in the upper trachea. Investigating the dynamics of the viral spread in vivo by N-protein analysis revealed significant differences: the B.1.1 and Delta infections were more pronounced in the alveolar space infecting the bronchial epithelium. In contrast, Omicron sporadically infected bronchial epithelial cells and was found mainly in the periphery of bronchi/bronchioles. Viral RNA load in collected oral swab peaked at 1 d.p.i. in B.1.1 and Delta infection and showed only slow decay in 7 days, while Omicron peaked during the 2–3 d.p.i. exceeding other variants and decayed rapidly after^2^ (Fig. 1c). The increased viral load in the oral swab of infected individuals may be related to the higher transmissibility of Omicron, but further investigation is needed to confirm this supposition.
